# Uterine Cavity Parameters Evaluated by Hysteroscopy can Predict the Live Birth Rate For Intrauterine Adhesion Patients

**DOI:** 10.3389/fmed.2022.926754

**Published:** 2022-06-17

**Authors:** Xingping Zhao, Dan Sun, Aiqian Zhang, Huan Huang, Xiuting Zhu, Shuijing Yi, Dabao Xu

**Affiliations:** Department of Gynecology, Third Xiangya Hospital of Central South University, Changsha, China

**Keywords:** intrauterine adhesion (IUA), uterine cavity, prognosis, live birth rate, hysteroscopic adhesiolysis

## Abstract

We aim to establish an objective and accurate prediction model by evaluating the uterine cavity and correlate these key factors with the live birth rate after hysteroscopic adhesiolysis (HA). A total of 457 intrauterine adhesions (IUA) patients were retrospectively enrolled in this study. The participants underwent HA and second-look hysteroscopy at the Third Xiangya Hospital of Central South University. Pregnancy outcomes, including spontaneous live births and no live births (miscarriages and infertility), were followed. Clinical parameters, containing the number of visible uterine horns and tubal ostia, the length of the uterine cavity, among others, were measured and analyzed to determine the dominant variables in an attempt to establish the live birth rate, prediction models. Women in the no live birth group were older than that in the live birth group (*P* = 0.0002, OR = 0.895, 95% CI: 0.844–0.949) and were more likely to be 2 gravidity (*P* = 0.0136, OR = 2.558, 95% CI: 1.213–5.394). Uterine cavity length in pre-HA hysteroscopy was longer in the live birth group (*P* = 0.0018, OR = 1.735, 95% CI: 1.227–2.453), and adhesion scores in pre-HA hysteroscopy were more frequently above 6 (*P* = 0.0252, OR = 0.286, 95% CI: 0.096–0.856) in the no live birth group. During the second-look, hysteroscopy, visible bilateral fallopian tube ostia were more frequently observed in the live birth group (*P* = 0.0339, OR = 11.76, 95% CI: 1.207–114.611), and adhesion scores were 4–6 (*P* < 0.0001, OR = 0.032, 95% CI: 0.007–0.146) and above 6 (*P* < 0.0001, OR = 0.012, 95% CI: 0.002–0.073) in the no live birth group. The areas under the curves (AUCs) of the pre-HA and second-look hysteroscopy prediction models were 0.7552 and 0.8484, respectively. We established an objective and accurate method for evaluating the uterine cavity by hysteroscopy, and second-look hysteroscopy is more valuable than the fist hysteroscopy in predicting the live birth rate following HA. Visible bilateral fallopian tube ostia or adhesion scores were <4 in the second-look hysteroscopy might predict live birth after surgery.

## Introduction

Intrauterine adhesions (IUA) are fibrous connective tissue covering the inner wall of the uterine cavity. With the widespread use of hysteroscopy in IUA patients, the reported incidence and diagnosis of IUA have increased significantly, with an average of 2.2 and 36.8%, respectively (1, 2), among women of childbearing age. The main risk factors related to IUA are pregnancy-related, and more than 90% of IUA are closely associated with curettage (3–5). The main clinical symptoms of IUA include menstrual abnormalities, recurrent miscarriages, and secondary infertility/subfertility. These symptoms may have adverse effects on subsequent fertility (6) and pregnancy outcomes. The hypothetical underlying mechanisms for poor reproductive outcomes are obstruction of sperm transport through the cervical canal, impaired embryo migration in the uterine cavity, or the implantation of embryos in the endometrium (7). Various studies have determined several factors that may affect reproductive outcomes (8, 9), including the extent, location (10), and severity of the adhesions. However, no specific feature is considered dominant. Hysteroscopic adhesiolysis (HA) is the current surgical treatment of choice for IUA. The main purpose of the HA is to restore the volume and shape of the uterine cavity and improve fertility potential (11), but the procedure can be challenging depending on the factors above (1).

Multiple methods of classifications of IUA have been proposed to classify the severity of IUA, and they are mostly based on hysteroscopic findings or hysterosalpingography. However, no classification system has yet been universally accepted, making the prognosis of IUA difficult to predict (11).

Visualization of the fallopian tube ostia, uterine mobility, and a normal uterine cavity size influence pregnancy outcomes. Comprehensive management, such as the placement of an intrauterine device (IUD) and/or a Foley catheter balloon, hyaluronic acid gel, amnion grafts insertion, hormonal therapy, or an early second-look hysteroscopy, usually supplement HA in an aim to restore uterine morphology and improve reproductive outcomes (12–14). The reproductive outcomes of patients who conceived after HA were significantly improved compared to previously used methods such as curettage or blind division of adhesions (3).

With the help of hysteroscopy, including pre-HA and second-look hysteroscopy, we aim to establish an objective and accurate method for evaluating the uterine cavity and correlate these critical factors with the live birth rate following HA as a prediction model. Moreover, we will compare the prediction models between pre-HA hysteroscopy and second-look hysteroscopy to determine the better model for clinical application.

## Materials and Methods

### Patients

All the 457 patients with moderate to severe IUA underwent HA and second-look hysteroscopy were retrospectively enrolled in this study. All the operations were conducted at the Third Xiangya Hospital of Central South University between February 2016 and May 2017. The ethics committee of the Third Xiangya Hospital of Central South University approved the study (IRB No.I-21053), and written informed consent for hysteroscopic surgery was signed voluntarily by each patient. During the two operations, each patients were scored by the same surgeon depending on the American Fertility Society (AFS) classification scoring system (15).

The inclusion criteria included: (1) IUA was diagnosed by hysteroscopy; (2) desire for fertility; (3) normal ovarian function and ovulation. The exclusion criteria were as follows: (1) endometrial tuberculosis resulting in IUA; (2) presence of other lesions such as endometrial polyps, uterine submucous myoma, uterine mediastinum, chronic endometritis, atypical hyperplasia, or endometrial malignancy; (3) patients with severe adhesion and unable to separate normal uterine cavity after hysteroscopy; (4) patients whose uterine cavity volume was too large or too small to insert IUD; (5) lost to follow-up; (6) participants who sufferd from postoperative infection; (7) patients had intrauterine surgery except curettage.

A total of 457 patients met the criteria and were enrolled in the study. All participants were followed up for more than 2 years after hysteroscopy. Pregnancy outcomes, including spontaneous miscarriages, live births, and infertility, were followed up. Medical records, operative reports, and hysteroscopic videos of the patients were reviewed.

### Surgical Methods and Postoperative Follow-Up Hysteroscopy

HA and second-look hysteroscopy were performed within 3 to 7 days after menstruation, with the patients took the lithotomy position and received intravenous anesthesia. The operations were performed in the operating room of the inpatient department in the form of ambulatory surgery. Patients fasted for 6 to 8 h before surgery. The distention media was sterile saline. The average intrauterine pressure was 110 mmHg and the average flow rate of distention media was 400 ml/min set by suction irrigation instrument (KARL STORZ SE & Co. KG–Tuttlingen, Baden-Württemberg, Germany). Hysteroscopy was carried out using a hysteroscope with an outer sheath diameter of 5.4 mm and a 5-Fr working channel (KARL STORZ SE & Co. KG–Tuttlingen, Baden-Württemberg, Germany). A blunt spreading dissection technique (16) and the cold scissors plowing technique (12) were used to reconstruct the normal uterine cavity.

After HA, a uterine-shaped stainless-steel IUD was inserted into the uterine cavity. The 12-Fr Foley catheter with the top catheter portion beyond the balloon removed was kept inside the uterine cavity and distended using 2.5 mL of sterile saline, with the balloon in the center of the IUD. Three milliliter hyaluronic acid gel was then injected into the uterine cavity through the channel of Foley catheter (17). A probe was used to measure the length of the uterine cavity after surgery.

The patients with moderate adhesion were placed with 12-Fr Foley catheter balloon for 3 days; the patients with severe adhesion were placed for 7 days; if the adhesion of the lower part of the internal orifice of the cervix was closed, left it for 3 weeks; if the lower part of the internal orifice of the cervix was partially closed, the balloon was left for 1 week; if the AFS score was <4, 12-Fr Foley cathether and IUD were no longer used. From the 5th to 25th day of menstruation, estradiol valerate was taken orally twice a day, 2 mg each time, and progesterone was added orally once a day, 0.2 g each time in the last 6 days.

Postoperatively, the second-look hysteroscopy follow-up strategy was conducted for all the patients in the 3 months after the first HA. During the follow-up procedure, hysteroscopy videos were recorded too. The operation methods, location of operation and anesthesia methods were the same as those of the first hysteroscopy. After the second-look hysteroscopy, the balloon was no longer used and the IUD was removed. The length of the uterine cavity was measured by probe again post-HA. After the second-look hysteroscopy, hormone drugs were no longer used, and patients were instructed to try pregnancy.

According to the AFS classification of IUA, the type of adhesion was rated as 1, 2, and 4 scores for filmy, filmy and dense, and dense, respectively. The extent of cavity involved was rated as 1, 2, and 4 scores for less than one third, one third to two-thirds, and more than two-thirds, respectively. The degree of adhesion was assessed by combining scores for the type of adhesion and the extent of the cavity involved. Other parameters, containing the number of visible uterine horns and tubal ostia, and the length of the uterine cavity were measured and recorded. Two gynecologists confirmed all data.

### Statistical Analysis

Statistical analysis was carried out with the Statistical Analysis System 9.4 (SAS Institute, North Carolina, USA). Differences between the two groups were tested using a chi-squared test or Fisher's exact test. Logistic regression analysis was used to determine the dominant variables for establishing the live birth rate prediction models. The areas under the curves (AUCs) of the models were compared to verify their prediction accuracy. A value of two-sided *P* < 0.05 was considered statistically significant.

## Results

Among the 457 patients, 231 had live births, 82 had a recurrent spontaneous abortion without live births, and 144 patients remained infertile at the end of 2 years, completing our study. Variables including age (*P* = 0.0002), previous HA history (*P* = 0.0002), menstrual flow (*P* = 0.0457), uterine cavity length (*P* = 0.0016 and *P* = 0.0095 in pre-HA and second-look hysteroscopy, respectively), number of visible uterine horns (*P* = 0.012 and *P* = 0.0255 in pre-HA and second-look hysteroscopy, respectively), tubal ostia (*P* = 0.0004 and *P* = 0 in pre-HA and second-look hysteroscopy, respectively), and adhesion scores (*P* = 0 and *P* = 0 in pre-HA and second-look hysteroscopy, respectively) were all significantly related to the live birth rate post-HA. Other variables, including gravidity history and disease duration, did not have any statistical significance related to the live birth rate post-HA (*P* > 0.05) (Table 1).

**Table 1:** Clinical characteristics of the patients with intrauterine adhesions.

**Clinical characteristics**	**Category**	**Live birth group**	**No live birth group**	***P*-value**
Age (y)	N (N missing)	231 (0)	226 (0)	0.0002
	Mean (SD)	30.3 (4.30)	31.9 (4.94)	
Gravidity	1	47 (20.3%)	53 (23.5%)	0.1767
	2	73 (31.6%)	41 (18.1%)	
	≥3	111 (48.1%)	132 (58.4%)	
Parity	1	228 (98.7%)	218 (96.5%)	0.1202
	2	2 (0.9%)	7 (3.1%)	
	≥3	1 (0.4%)	1 (0.4%)	
Surgical abortion	1	75 (32.5%)	75 (33.2%)	0.3124
	2	75 (32.5%)	53 (23.5%)	
	≥3	81 (35.1%)	98 (43.4%)	
Recurrent IUA	Yes	42 (18.2%)	75 (33.2%)	0.0002
	No	189 (81.8%)	151 (66.8%)	
Menstruation	Eumenorrhea	29 (12.6%)	26 (11.5%)	0.0457
	Oligomenorrhea	189 (81.8%)	170 (75.2%)	
	Amenorrhea	12 (5.2%)	29 (12.8%)	
Uterine cavity length in pre-HA (cm)	Mean (SD)	7.3 (0.72)	7.0 (0.84)	0.0016
Visibility of uterine horn in pre-HA	Bilateral invisible	19 (8.2%)	33 (14.6%)	0.0120
	Unilateral invisible	26 (11.3%)	33 (14.6%)	
	Bilateral visible	186 (80.5%)	160 (70.8%)	
Visibility of fallopian tube ostia in pre-HA	Bilateral invisible	29 (12.6%)	52 (23.0%)	0.0004
	Unilateral invisible	34 (14.7%)	44 (19.5%)	
	Bilateral visible	168 (72.7%)	130 (57.5%)	
Adhesion scores in pre-HA	≤ 2	14 (6.1%)	8 (3.5%)	0.0000
	2–4	91 (39.4%)	36 (15.9%)	
	4–6	85 (36.8%)	68 (30.1%)	
	>6	41 (17.7%)	100 (44.2%)	
Uterine cavity length in second-look hysteroscopy (cm)	Mean (SD)	7.2 (0.52)	7.0 (0.68)	0.0095
Visibility of uterine horn in second-look hysteroscopy	Bilateral invisible	4 (1.7%)	10 (4.4%)	0.0255
	Unilateral invisible	2 (0.9%)	6 (2.7%)	
	Bilateral visible	225 (97.4%)	210 (92.9%)	
Visibility of fallopian tube ostia in second-look hysteroscopy	Bilateral invisible	5 (2.2%)	22 (9.7%)	0.0000
	Unilateral invisible	5 (2.2%)	23 (10.2%)	
	Bilateral visible	221 (95.7%)	181 (80.1%)	
Adhesion scores in second-look hysteroscopy	≤ 2	39 (16.9%)	3 (1.3%)	0.0000
	2–4	152 (65.8%)	56 (24.8%)	
	4–6	35 (15.2%)	109 (48.2%)	
	>6	4 (1.7%)	44 (19.5%)	

*IUA, intrauterine adhesion; HA, hysteroscopic adhesiolysis*.

The risk factors for live birth rate were analyzed by univariate analysis. A multivariate logistic regression analysis was carried out based on the meaningful variables (*P* < 0.05) found by univariate analysis. Compared with the live birth group, patients in the no live birth group were older (*P* = 0.0002, OR = 0.895, 95% CI: 0.844–0.949) and were more likely to be pregnant twice (*P* = 0.0136, OR = 2.558, 95% CI: 1.213–5.394). Uterine cavity length in pre-HA hysteroscopy was longer in the live birth group (*P* = 0.0018, OR = 1.735, 95% CI: 1.227–2.453), and the adhesion scores in pre-HA hysteroscopy were more frequently above 6 (*P* = 0.0252, OR = 0.286, 95% CI: 0.096–0.856) in the no live birth group. During the second-look, hysteroscopy, visible bilateral fallopian tube ostia were more frequently observed in the live birth group (*P* = 0.0339, OR = 11.76, 95% CI: 1.207–114.611), and adhesion scores were 4–6 (*P* < 0.0001, OR = 0.032, 95% CI: 0.007–0.146) and above 6 (*P* < 0.0001, OR = 0.012, 95% CI: 0.002–0.073) in the no live birth group (Table 2).

**Table 2:** Logistic regression analysis of the risk factors for live birth rate.

**Variables**	**Category**	**Univariate analysis**		**Multivariate analysis**	
		**OR (95% CI)**	***P*-value**	**OR (95% CI)**	***P*-value**
Age		0.929 (0.893–0.968)	0.0004	0.895 (0.844–0.949)	0.0002
Gravidity	1				
	2	2.008 (1.16–3.474)	0.0127	2.558 (1.213–5.394)	0.0136
	≥3	0.948 (0.595–1.512)	0.8235	1.706 (0.882–3.297)	0.1122
Parity	1				
	2	0.273 (0.056–1.329)	0.108		
	≥3	0.956 (0.059–15.382)	0.9748		
Abortion	1				
	2	1.415 (0.88–2.277)	0.1524		
	≥3	0.827 (0.535–1.277)	0.3905		
Recurrent IUA	Yes				
	No	2.235 (1.448–3.45)	0.0003		
Menstruation	Eumenorrhea				
	Oligomenorrhea	0.997 (0.565–1.76)	0.9911		
	Amenorrhea	0.371 (0.158–0.874)	0.0233		
Uterine cavity length in pre-HA		1.550 (1.197–2.007)	0.0009	1.735 (1.227–2.453)	0.0018
Visibility of uterine horn in pre-HA	Bilateral invisible				
	Unilateral invisible	1.368 (0.638–2.936)	0.4207		
	Bilateral visible	2.019 (1.105–3.689)	0.0223		
Visibility of fallopian tube ostia in pre-HA	Bilateral invisible				
	Unilateral invisible	1.386 (0.732–2.622)	0.3162		
	Bilateral visible	2.317 (1.393–3.854)	0.0012		
Adhesion scores in pre-HA	≤ 2				
	2-4	1.444 (0.558–3.737)	0.4483	1.960 (0.643–5.975)	0.2368
	4-6	0.714 (0.283–1.802)	0.4760	1.067 (0.37–3.08)	0.9042
	>6	0.234 (0.091–0.601)	0.0025	0.286 (0.096–0.856)	0.0252
Uterine cavity length in second-look hysteroscopy		1.497 (1.092–2.052)	0.0122		
Visibility of uterine horn in second-look hysteroscopy	Bilateral invisible				
	Unilateral invisible	0.833 (0.115–6.013)	0.8565		
	Bilateral visible	2.679 (0.827–8.671)	0.1002		
Visibility of fallopian tube ostia in second-look hysteroscopy	Bilateral invisible				
	Unilateral invisible	0.957 (0.243–3.766)	0.9493	4.300 (0.345–53.62)	0.2572
	Bilateral visible	5.372 (1.995–14.467)	0.0009	11.760 (1.207–114.611)	0.0339
Adhesion scores in second-look hysteroscopy	≤ 2				
	2–4	0.209 (0.062–0.703)	0.0114	0.232 (0.053–1.023)	0.0536
	4–6	0.025 (0.007–0.085)	<0.0001	0.032 (0.007–0.146)	<0.0001
	>6	0.007 (0.001–0.033)	<0.0001	0.012 (0.002–0.073)	<0.0001

*IUA, intrauterine adhesion; HA, hysteroscopic adhesiolysis*.

**Figure 1:**
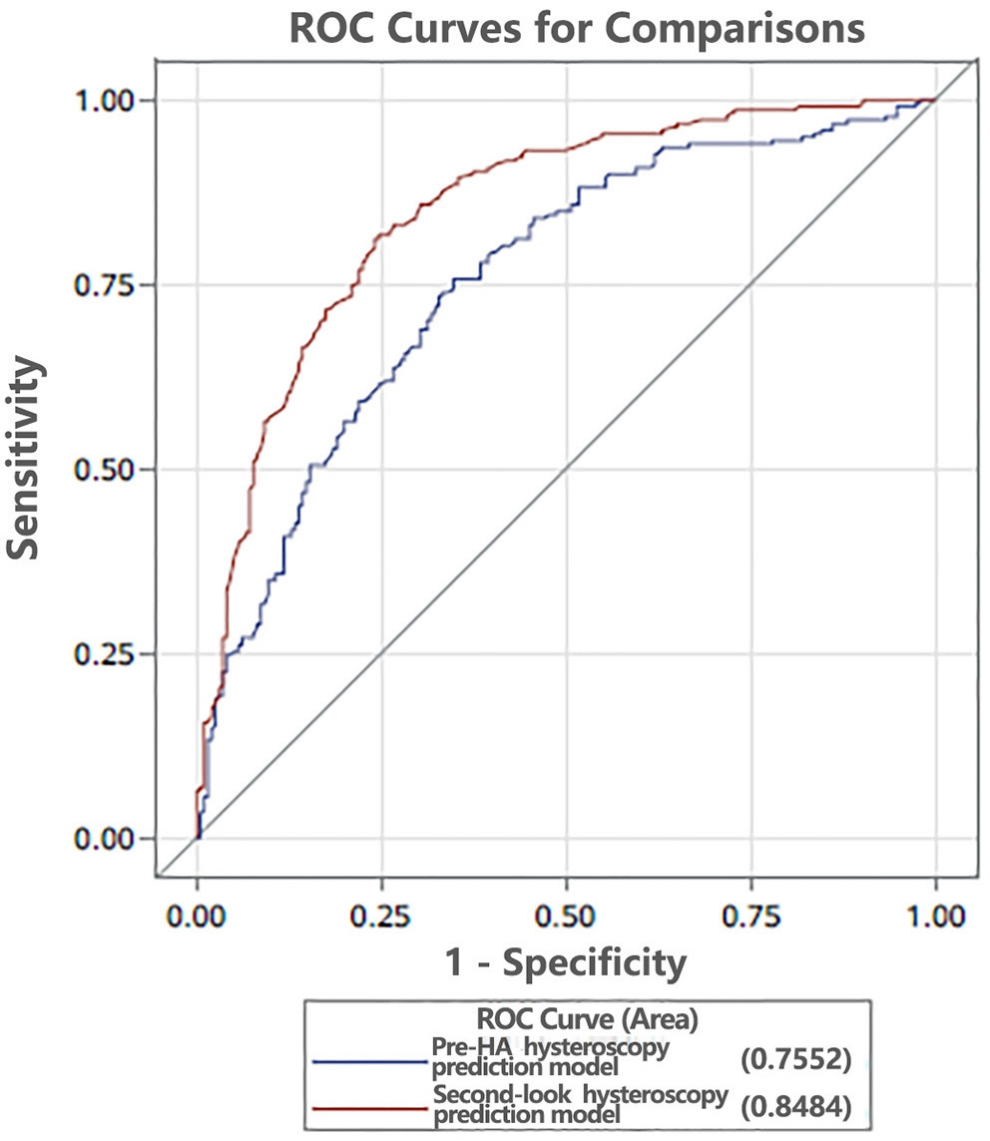
The areas under the curves (AUCs) of the pre-HA and second-look hysteroscopy prediction models were 0.7552 and 0.8484, respectively. HA, hysteroscopic adhesiolysis.

Bivariate and binary logistic regression analysis revealed that pre-HA and second-look hysteroscopy parameters were closely related to the live birth rate in IUA patients. The AUCs of the pre-HA and second-look hysteroscopy prediction models were 0.7552 and 0.8484, respectively (Figure 1). There was a significant difference in AUCs between models of pre-HA and second-look hysteroscopy in the prediction of the live birth rate in patients with IUA (*P* < 0.0001) (Table 3), and the model of second-look hysteroscopy showed excellent performance in the prediction of the live birth rate of IUA patients after HA.

**Table 3:** Comparison of the areas under the curves of the prediction models.

**Comparison of AUCs**	**Estimate**	**Std. error**	**95%CI**	*X*2^*^	Pr > *X*2^*^
Model of second-look hysteroscopy – Model of pre-HA	0.0932	0.0223	0.0495-0.1369	17.4953	<0.0001

AUCs, areas under the curves; *, chi-square test for the entire group; HA, hysteroscopic adhesiolysis.

## Discussion

The main concern of patients with IUA is their chance of having a post-HA live birth. HA aims to reconstruct the volume and shape of the uterine cavity and also improves fertility potential. In our study, IUA was treated with the “cold scissors plowing technique” (12) until the entire uterine cavity had returned to normal with clearly visible bilateral fallopian tube ostia. The prognosis after HA was assessed and reviewed during the second-look hysteroscopy to predict the likelihood of IUA recurrence, including whether the signs and symptoms improved, worsened, or remained unchanged after HA and the various factors affecting pregnancy outcomes.

Several methods have been proposed to predict the live birth rate for patients with IUA after undergoing HA (3, 4, 8, 15, 17–19). The AFS scoring system is the most commonly used clinical evaluation scale for the severity of IUA and helps predict pregnancy outcomes. The variables of the AFS scoring system include the type of adhesion, the extent of the cavity involved, and menstrual status. Menstrual status is used to evaluate the endometrial function, but this variable is subjective and may not accurately evaluate endometrial function. In our study, variables including age, previous HA history, uterine cavity length, number of visible uterine horns and tubal ostia, and adhesion scores were closely related to the post-HA's live birth rate. Other parameters, including disease duration and gravidity history, did not have any statistical significance related to post-HA's live birth rate.

The extent of IUA has been considered as an important factor relating to the reproductive outcome, and it has already been included in most classification systems. HA appears to be indicated when the extent of the adhesion is moderate to severe or when access to tubal ostia is blocked (5). The smaller proportion of the affected area was associated with the better the prognosis of HA.

The location of IUA is associated with the risk of postoperative adhesion reformation. The risk of IUA recurrence will increase when adhesions are located at the uterine cornua, specifically at the tubal ostium (19). Our study's visualization of the uterine cornua and fallopian tube ostia was closely related to pregnancy outcomes. Therefore, clear cornual angles and visualization of fallopian tube ostia are considered important prognostic factors for successful surgery.

Reproductive outcomes correlate with the severity of the initial adhesions (5). According to the American Society of Reproductive Medicine (ASRM), the type and severity of the adhesions correlate with the 2 pregnancy outcomes: (1) patients have 70 to 80% full-term pregnancy success rates, and normal menstruation is restored in over 90% of patients after removing mild to moderate uterine adhesions (8, 20); (2) if the IUA is severe or causes extensive damage to the endometrial lining, full-term pregnancy success rates are only 20 to 40% after treatment (20). In our study, scores of the type of adhesion and the extent of the cavity involved were the main factors affecting pregnancy outcome.

Although the prognosis of women suffering from IUA is steadily improving, it is still far from ideal. Outcomes of present-day treatment are excellent in relieving and correcting menstrual disorders but far from satisfactory in terms of restoration of fertility. Successful treatment of IUA depended on complete separation of adhesive tissue and the prevention of adhesion recurrence, as the reproductive outcome is adversely affected by the frequent recurrence of adhesions (21). This situation may equivalent to the fact that the pathological state in these cases is caused by the IUA and the replacement of the endometrium by connective tissue.

Moreover, some endometrial injuries can be irreparable. The damage may be very severe, leading to treatment failure. Therefore, the desired results are not achieved, and the treatment has to be abandoned. Restoration of menstruation or even fertility after treatment of IUA does not necessarily indicate a return to normal, as the ensuing pregnancy may be subject to several complications. The high rates of abortion (25%) and premature labor (9%) are probably caused by the endometrial dysfunction, despite its anatomic restoration (22). In this study, estrogen and progesterone were used to promote endometrial growth during the 3 months interval between the two hysteroscopy giving sufficient time for endometrial repair. Therefore, we didn't advise patients to try pregnancy after the pre-HA, but wait for the second-look hysteroscopy considering the second-look hysteroscopy can provide a better uterine environment for the implantation of embryo. The results of this study show that, compared with the prediction model of pre-HA hysteroscopy, the prediction model of the second-look hysteroscopy provides a better prediction of the live birth rate for patients with IUA following HA. The results also revealed that the situation of uterine cavity observed in the second-look hysteroscopy was further improved, which was more closely related to the pregnancy outcome.

In conclusion, we established an objective and accurate method for evaluating the uterine cavity by hysteroscopy, and second-look hysteroscopy plays a certain role in predicting the live birth rate following HA. Visible bilateral fallopian tube ostia or adhesion scores were <4 in the second-look hysteroscopy might predict live birth after surgery, which still needs to be confirmed by a large sample of prospective studies.

## Data Availability Statement

The raw data supporting the conclusions of this article will be made available by the authors, without undue reservation.

## Ethics Statement

The studies involving human participants were reviewed and approved by the Ethics Committee of the Third Xiangya Hospital of Central South University. The patients/participants provided their written informed consent to participate in this study.

## Author Contributions

DX and SY conceived and designed the study. DS and XZha drafted the manuscript and analyzed the data. AZ and HH were responsible for the picture and article format. XZha and XZhu reviewed the data. All authors contributed to the article and approved the submitted version.

## Funding

This study was supported by the Natural Science Foundation of Changsha (kq2202424), the Natural Science Foundation of Hunan Province (2021JJ40956), the National Key Research and Development Program of China (2018YFC1004800), the Hunan Provincial Clinical Medical Technology Innovation Guiding Project (2020SK53605 and 2020SK53606), and the Natural Science Foundation of Hunan Province (2021JJ40593).

## Conflict of Interest

The authors declare that the research was conducted in the absence of any commercial or financial relationships that could be construed as a potential conflict of interest.

## Publisher's Note

All claims expressed in this article are solely those of the authors and do not necessarily represent those of their affiliated organizations, or those of the publisher, the editors and the reviewers. Any product that may be evaluated in this article, or claim that may be made by its manufacturer, is not guaranteed or endorsed by the publisher.
